# On to Des Moines

**Published:** 1895-07

**Authors:** 


### EDITORIAL.

### ON TO DES MOINES.

In two short months the active, progressive veterinarians of the United States will have turned their faces toward Des Moines to take part in what now promises to be the most important meeting of the United States Veterinary Medical Association. It goes to Des Moines at this time for many reasons. First, and an all-important one, is, that for the first time it will be said of our organization that it went West for its annual gathering. While we have been to Cincinnati and Chicago, it has oft-times been repeated that these cities could hardly be longer considered as Western cities, and that many of the members who met with us at Chicago traveled as far and longer distances west of the Windy City than we in the extreme East had done to reach Chicago. Another reason, better and stronger than this, that we go at the solicitation and wishes of a large body of the profession in the Missouri and Mississippi Valleys, who are among the most progressive and aggressive members of the profession in our land, and Iowa to-day stands at the head of these several States, a wonderfully strong centre of Association activity. We go at this time to join hands with our Western colleagues at the broadest territorial solicitation ever filed in the history of our organization as a plea for the place of meeting. We will there receive the heartiest welcome and most sincere reception that it has been our lot to receive west of the Alleghenies, and we will there sink forever the lingering reflections that we were not a national organization save in name. It will be a final step to decide whether we shall realize that long hope, whose conception and birth attained announcement through that ever-faithful, zealous member and officer, our honored Treasurer, "Shall we go to California in 1897?" Des Moines, in 1895, will decide the Mecca of '97, and California has already spoken her heartfelt desire and wishes, and sends to us in September her delegate, a representative veterinarian among her many worthy members of our profession. She sends to mingle with us one of her young men, a strong and faithful Association veterinarian, and whose zeal and interest in the profession is as broad and deep as the State he will represent is rich in earnest, strong, professional votaries.

We go to Des Moines at a peculiar epoch in the history of the growth of our profession, just as it is emerging from one of the most trying crises in our national history, and just on the eve of a new era of activity, following the most severe slump in values of horses, our chief support in general practice, that this or any other country ever witnessed, but which time will prove held many blessings in disguise for us.

We will there meet to consider questions of the most stupendous importance to our people and to ourselves. The future of the veterinarian lies almost completely in the field of sanitary science, and this means all-important questions for us to decide in the best methods, the most economical plans, the greatest safety to our people, and the highest preservation of the health and commercial values of the great interests we represent and guard. It reaches further, it touches our influence and power in National, State, City, Town, and Borough legislation, that only wise laws may be adopted and executed on these all-important questions. It speaks to us in no uncertain tones as to the deep and earnest interest we must continue to take in our veterinary educational institutions and the helping hand we must give them that their usefulness and worth may be extended to a wider sphere among our people.

Aside from these great responsibilities that will be considered, we go to participate in one of the most attractive programmes it has been our lot to consider for many years—a programme containing the names of Huidekoper, Schwarzkopf, Harger, Williams, Reynolds, Tait Butler, L. McLean, Trumbower, Niles, and others, each with papers of special importance. We, therefore, appeal to the veterinarians of the States to lay aside the duties of the hour and bend every effort to meet with us at this all-important time; to come and help us discuss and solve these great questions of the day. To our professional friends across the border, some of whom are in our ranks, we send the warmest expressions of our wishes to come and join with us, that we may have the pleasure of your presence, the value of your advice in our discussions, and that the profession may be more of a unit in all that it does on all these great questions of universal interest, wrapped up in our food supply, our professional existence, our national importance among the great army of busy workers.

